# Co-Produced for Use: Developing an Information and Symptom Self-Management Resource for People With Functional Neurological Disorder (FND)

**DOI:** 10.1192/bjo.2022.332

**Published:** 2022-06-20

**Authors:** Jai Ramchandani, Cameron Manson, Amina Mushtaq, Verity Williams, Maxwell Pickard, Alan Dunlop, Rafey Faruqui

**Affiliations:** 1 King's College London, London, United Kingdom; 2 Kent and Medway NHS and Social Care Partnership Trust, Kent and Medway Medical School, Canterbury, United Kingdom; 3 Kent and Medway NHS and Social Care Partnership Trust, Thanet, United Kingdom

## Abstract

**Aims:**

Patients with Functional Neurological Disorder (FND) often endure low quality of life. Understanding the diagnosis is critical to management, but patients with FND do not always receive appropriate information about their condition. The patient journey through healthcare services can be complex, with often long waits for specialist attention. Creating psychoeducation resources for patients is important to improve patient experience and outcomes. We developed a symptom self-management patient education booklet with an FND symptom recording template, using a co-production model, in a community neuropsychiatry setting.

**Methods:**

We used co-production as part of a quality improvement project (QIP) at East Kent Neuropsychiatry Service, to produce a patient education booklet with symptom self-management information and a symptom recording template. The QIP cycle involved input from 11 participants. Initially, 3 medical students and 4 multi-disciplinary team members adapted an existing booklet, removing medical jargon and simplifying diagrams. The adapted booklet was distributed to patients with FND who were attending psychoeducation/Cognitive Behavioural Therapy group sessions. One week later, four patients discussed the booklet with a medical student facilitator; both quantitative and qualitative feedback was obtained. Feedback was gathered using an adapted 20 point Ensuring Quality of Information for Patients (EQIP) tool. Patient responses were recorded, and qualitative themes identified.

**Results:**

Four themes were found from qualitative feedback during co-production: need for a glossary; an expanded resource list; more diagrams to simplify text; and for the booklet to also address family, friends, and carers. The EQIP questionnaire feedback emphasised that the booklet contained too much medical jargon and that it didn't personally address the reader. On average patients scored the booklet 53.33% using the EQIP questionnaire.

The booklet was further adapted and a glossary, further diagrams and a section addressing family, friends, and carers was added. Further resources were added and the text was simplified for clarity.

**Conclusion:**

This QIP shows the value of co-producing information for an underserved patient population. Patient psychoeducation is a key part of treatment; involving patients at an early stage of the development of information and self-management tools will increase their acceptability to patients and improve the accessibility of patient psychoeducation.

